# Circulating exosomes from patients with systemic lupus erythematosus induce an proinflammatory immune response

**DOI:** 10.1186/s13075-016-1159-y

**Published:** 2016-11-16

**Authors:** Joo Youn Lee, Jin Kyun Park, Eun Young Lee, Eun Bong Lee, Yeong Wook Song

**Affiliations:** 1 Department of Molecular Medicine and Biopharmaceutical Sciences, BK 21 plus Graduate School of Convergence Science and Technology, and College of Medicine, Medical Research Institute, Seoul National University, Seoul, Korea; 2 Division of Rheumatology, Department of Internal Medicine, Seoul National University College of Medicine, Daehak-ro, Jongno-gu, Seoul, 03082 Korea

**Keywords:** Systemic lupus erythematosus, Exosome, Cytokine, Inflammation, Toll-like receptor

## Abstract

**Background:**

Exosomes are involved in intercellular communication. The aim of this study was to investigate whether circulating exosomes effectively contribute to the inflammatory response in systemic lupus erythematosus (SLE).

**Methods:**

Exosomes were purified from SLE patients and healthy controls (HCs). Healthy peripheral blood mononuclear cells (PBMCs) were stimulated with exosomes isolated from SLE patients and HCs in the presence or absence of Toll-like receptor (TLR) inhibitors. Production of interferon (IFN)-α, tumor necrosis factor (TNF)-α, interleukin (IL)-1β, and IL-6 were measured. Correlation between exosome levels and SLE disease activity was examined.

**Results:**

The serum exosomes levels were significantly higher in SLE patients than in HCs. SLE exosomes induced a higher production of IFN-α, TNF-α, IL-1β, and IL-6 compared to healthy exosomes. SLE serum that was depleted of exosomes and SLE exosomes that were mechanically disrupted failed to induce any significant cytokine production. Exosome-mediated production of TNF-α, IL-1β, and IL-6 was decreased by the TLR4 antagonist, whereas that of IFN-α was suppressed by the TLR1/2, TLR7, and TLR9 antagonists. Exosome levels correlated with disease activity in SLE patients (rho = 0.846, *p* = 0.008).

**Conclusions:**

The circulating exosomes are immunologically active and their levels correlate with disease activity in SLE patients. The circulating exosomes might serve as novel biomarkers of SLE disease activity.

**Electronic supplementary material:**

The online version of this article (doi:10.1186/s13075-016-1159-y) contains supplementary material, which is available to authorized users.

## Background

Effective communication between immune cells is critical for a coordinated immune response. In systemic lupus erythematosus (SLE), the dysfunctional communication leads to abnormal activation of immune cells, autoantibody production, complement activation, and tissue inflammation that ultimately damages multiple organs [1]. In addition to cytokines such as tumor necrosis factor (TNF)-α, interferons (IFNs), and interleukins (ILs), acellular microvesicles, delimited by a lipid bilayer, have been postulated as effective mediators of intercellular communication in both physiologic and pathologic situations [2]. Exosomes are extracellular vesicles, 50–100 nm in diameter, which originate from the multivesicular bodies of endosomes. They carry intergrins and tetraspanins such as CD63 and CD81 on their surface [3, 4]. Although the primary role of exosome production is thought to be the removal of unwanted cellular components, those components on and within exosomes can exert diverse biological functions depending on the cells of origin [5–8]. Exosomes are believed to contain molecules that can induce a proinflammatory response via pattern-associated molecular patterns (PAMP) receptors such as Toll-like receptors (TLRs). TLRs are a family of receptors through which the mammalian innate immune cells recognize invading pathogens to mount a proper immune response [9]. TLRs 1, 2, 4, 5, and 6 are expressed on the cell surface, whereas TLRs 3, 7, 8, and 9 are located in intracellular endosomes [10, 11]. Recently microRNAs (miRNAs) in the exosomes, which are released from cancer cells, have been shown to induce inflammatory responses via TLRs and to promote cancer metastasis [12].

Microparticles derived from SLE patients have been shown to have a higher concentration of immunoglobulins and complement components at the expense of cytoskeletal proteins as compared to those derived from healthy controls (HCs) [10, 11]. Accordingly, the inflammatory process of SLE might produce “SLE-specific” exosomes that might, in return, amplify the abnormal immune response. The aim of the current study was to investigate whether SLE exosomes are able to mount a significant inflammatory response and whether the levels of circulating exosomes correlate with SLE disease activity.

## Methods

### Patients

HCs (*n* = 8), and patients with SLE (*n* = 19) were enrolled after obtaining informed consent (Additional file 1: Table S1). The patients fulfilled the American College of Rheumatology revised criteria for the classification of SLE [13]. SLE disease activity index 2000 (SLEDAI-2K) was determined at the time of blood sampling [14].

### Exosome isolation and quantification

Serum was prepared from fresh peripheral blood by centrifugation. Exosomes were purified from serum using ExoQuick (System Biosciences, CA, USA) according to the manufacturer’s instructions. Briefly, 63 μL ExoQuick solution was added to 250 μL serum. After 30 min at 4 °C, exosomes were pelleted using centrifugation at 1500 g for 30 min. The pellets containing exosomes were resuspended in 50 μL sterile water.

Exosomes were quantified using EXOCET Exosome Quantification Assay Kit and ELISA of exosome surface markers (EXOEL-CD63A-1 and EXOEL-CD81A-1; System Biosciences) according to the manufacturer’s instructions. Of note, EXOCET measures enzymatic activity of acetylcholinesterases (AChE), which are enriched within exosomes. EXOEL-CD63A-1 and EXOEL-CD81A-1 are a direct enzyme-linked immunosorbent assay (ELISA)-based method to quantify CD63 and CD81.

### Exosome disruption

Exosomes were mechanically disrupted using ultra-sonication (Bioruptor, BMS Co., USA) for 15 min (10 s sonication and 30 s cooling cycle) on ice.

### Transmission electron microscopy (TEM)

Exosomes were dried onto a copper grid with a lacey carbon film. The grid was negatively stained with 2 % uranyl acetate and imaged with a Carl Zeiss LIBRA120 (Carl Zeiss, Oberkochen, Germany) with an accelerating voltage set to 120 kV. Images were taken with a Orius SC200W 2 CCD camera (Gatan Inc., CA, USA).

### Stimulation of immune cells with exosomes

Peripheral blood mononuclear cells (PBMCs) were isolated from heparinzed peripheral venous blood of HCs by density gradient centrifugation using Ficoll-paque plus gradient (GE Healthcare Biosciences, Uppsala, Sweden). Cell viability was assessed with trypan blue dye exclusion. PBMCs (5 × 10^6^ cells/mL) were stimulated with 10 μL purified exosomes in RPMI-1640 supplemented with 100 U/mL penicillin and 100 μL/mL streptomycin for 24 h at 37 °C in a 5 % CO_2_ incubator.

To investigate the involvement of TLRs, PBMCs were pretreated with TLR1/2 antagonist (CU-CPT22; Calbiochem, MA, USA), TLR4 antagonist (LPS-RS ultrapure; Invivogen, CA, USA), TLR7 antagonist (ODN20958; Invivogen, CA, USA), or TLR9 antagonist (ODN2088; Invivogen, CA, USA) for 1 h. Then the cells were incubated with exosomes for 24 h. Following stimulation, levels of IFN-α, TNF-α, IL-1β, and IL-6 in the supernatants were measured using ELISA according to the manufacturers’ instructions (IFN-α, PBL Assay Science, NJ, USA; TNF-α, IL-1β, and IL-6, BD Biosciences, CA, USA).

### Exosome uptake by cells

The purified exosomes were labeled with carboxyfluorescein succinimidyl diacetate ester (CFSE) using Exo-Glow kits (System Biosciences, CA, USA) according to the manufacturer’s instructions. PBMCs were incubated with the CFSE-labeled exosomes. After the indicated time points, PBMCs were stained and analyzed using FACSCalibur flow cytometer and Cellquest software (BD Biosciences).

For the confocal microscopy, PBMCs were treated with the CFSE-labeled exosomes and then stained with LysoTracker red DND-99 (Life Technologies, MA, USA), which marks endosomes. After cover-slipping with Fluoroshield with DAPI (ImmunoBioScience, WA, USA), the cells were imaged using a Leica TCS SP8 (Leica, Wetzlar, Germany).

For co-localization of exosomes and TLRs, PBMCs were treated with the CFSE-labeled exosomes and then were fixed in 4 % paraformaldehyde (Sigma, MO, USA) in phosphate-buffered saline (PBS). After washing, PBMCs were stained with antibodies against TLR2 (TL2.1, mouse monoclonal), TLR4 (HTA125, mouse monoclonal), TLR7 (4G6, mouse monoclonal), or TLR9 (26C593.2, mouse monoclonal). Of note, for TLR7 and TLR9 staining, the cells were permeabilized with 0.1 % triton X-100 (Sigma) prior to incubation with primary antibodies. All TLR antibodies were purchased from Life Technologies (MA, USA). Alexa Fluor 488 goat anti-mouse IgG (Molecular Probes, CA, USA) was applied in PBS containing 10 % fetal bovine serum (FBS). After cover-slipping with Fluoroshield with DAPI (ImmunoBioScience), co-localization of exosomes and TLRs were imaged using a Leica TCS SP8 (Leica).

### Statistical analyses

Differences between two groups were assessed by Mann-Whitney *U* tests or Wilcoxon matched-pairs signed rank test, as appropriate. The correlations between SLEDAI and cytokine production were examined using Spearman correlation. All reported *p* values were two-sided. *P* < 0.05 was considered to indicate statistical significance. All statistical analyses were performed using GraphPad Prism 5.01 (GraphPad Software Inc., CA, USA).

## Results

### Exosomes are enriched in serum of SLE patients

**Fig. 1:**
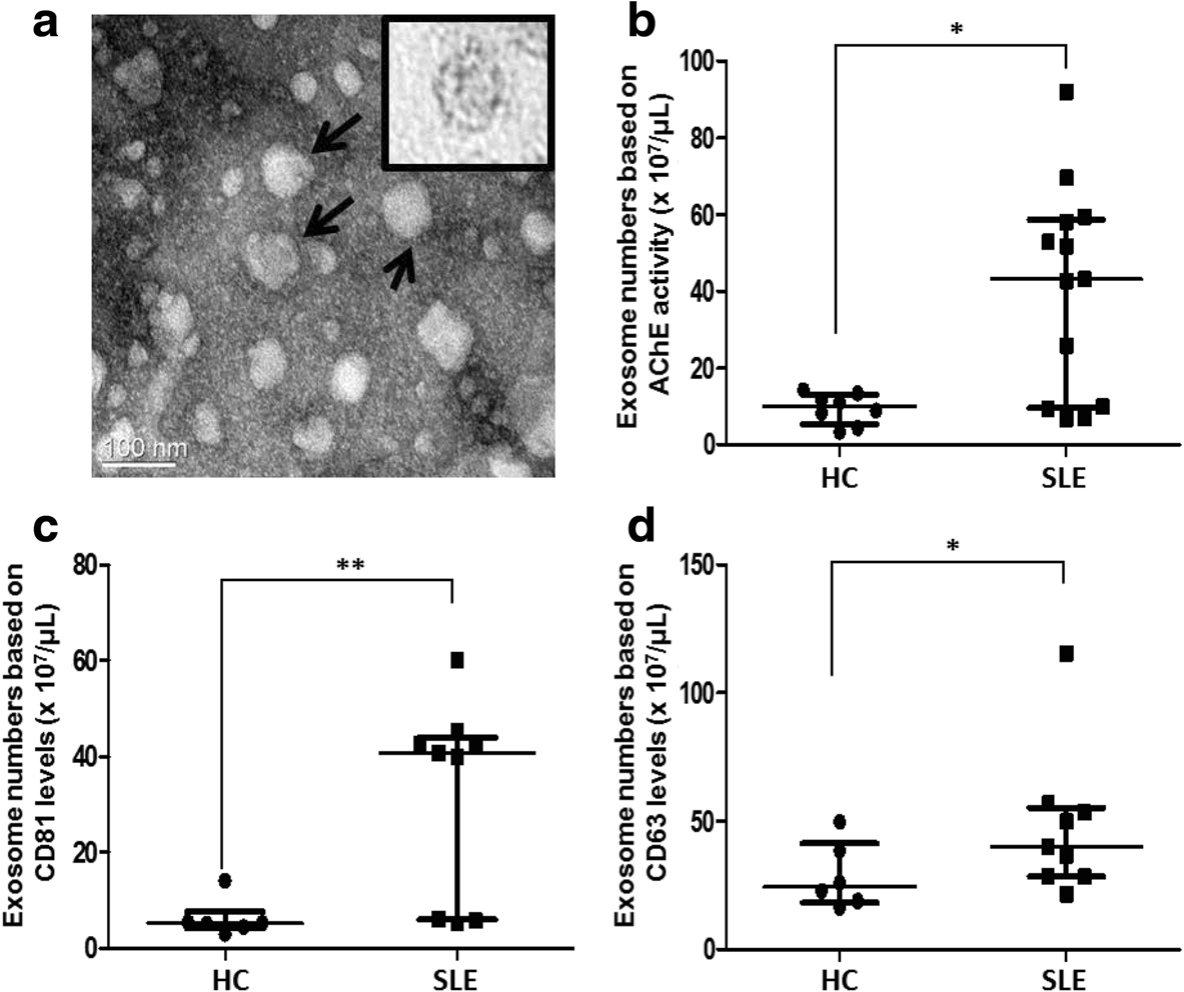
Enriched exosomes in SLE. **a** The size and shape of purified exosomes were evaluated by TEM imaging. The exosomes were 50–100 nm in size (*arrows*) and had a lipid layer (*insert*). **b** Systemic lupus erythematosus (*SLE*) patients had higher serum levels of exosomes than healthy controls (*HCs*) by acetylcholinesterase (*AChE*) assay. Both exosome protein markers CD81 (**c**) and CD63 (**d**) were higher in SLE than in HC. Data are presented as the median and interquartile range. *p < 0.05, **p < 0.01

The purified exosomes were visualized using TEM as shown in Fig. 1a. They were 50–100 nm in size. Since exosomes are rich in AChE [15, 16], CD63, and CD81 [17, 18], their levels can be used to estimate the number of exosome particles. Based on AChE assay, the serum levels of exosomes were significantly higher in SLE patients (*n* =13) than in HCs (*n* = 8) (median with interquartile range (IQR), μL: 43.10 × 10^7^ (9.61 × 10^7^–58.44 × 10^7^) vs. 10.07 × 10^7^ (5.36 × 10^7^–13.01 × 10^7^), respectively; *p* = 0.023) (Fig. 1b). CD81 levels were significantly higher in SLE exosomes (*n* = 9) than HC exosomes (*n* = 6) (median (IQR), μL: 40.80 × 10^7^ (6.01 × 10^7^–44.00 × 10^7^) vs. 5.30 × 10^7^ (4.14 × 10^7^–7.68 × 10^7^), respectively; *p* = 0.008) (Fig. 1c).

Similarly, CD63 levels were higher in SLE exosomes (*n* = 9) than HC exosomes (*n* = 6) (median (IQR), μL: 40.15 × 10^7^ (28.41 × 10^7^–55.29 × 10^7^) vs. 24.45 × 10^7^ (18.29 × 10^7^–41.30 × 10^7^), respectively; *p* = 0.013) (Fig. 1d). Taken together, the serum levels of circulating exosomes were significantly higher in SLE patients than in HCs.

### SLE exosomes induces stronger cytokine production

**Fig. 2:**
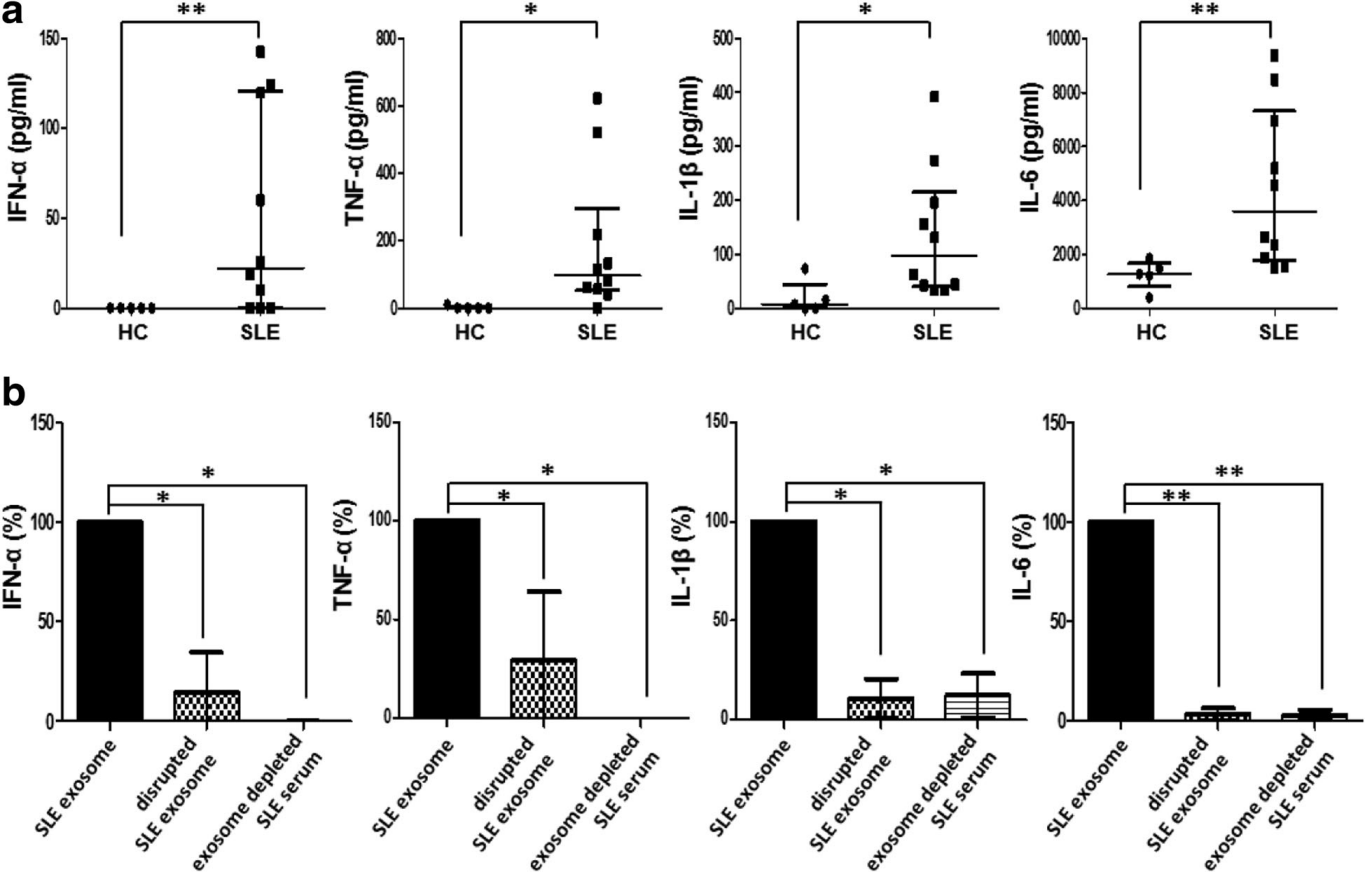
Cytokine production by exosomes. **a** Healthy PBMCs were stimulated with exosomes from healthy controls (*HC*) and systemic lupus erythematosus (*SLE*) patients, and production of IFN-α, TNF-α, IL-1β, and IL-6 were measured. Data are presented as the median and interquartile range. **b** Cytokine production by SLE exosomes that were mechanically disrupted and SLE serum that was depleted of exosomes relative to SLE exosomes were compared. Data are presented as the median and interquartile range. *p < 0.05, **p < 0.01. *IFN* interferon, *IL* interleukin, *TNF* tumor necrosis factor

PBMCs (5 × 10^5^ cells) from other additional healthy donors were treated with 10 μL of exosomes purified from HCs (*n* = 5) or SLE patients (*n* = 10). As compared to the HC exosomes, the SLE exosomes induced PBMCs to produce significantly more IFN-α (median (IQR), pg/mL: 0.00 (0.00–0.00) vs. 22.34 (0.00–120.9), respectively; *p* = 0.004), TNF-α (median (IQR), pg/mL: 0.00 (0.00–5.00) vs. 97.47 (53.17–294.9), respectively; *p* = 0.023), IL-1β (median (IQR), pg/mL: 7.10 (0.00–44.52) vs. 97.24 (40.20–215.2), respectively; *p* = 0.013), and IL-6 (median (IQR), pg/mL: 1259 (802.5–1669) vs. 3596 (1790–7352), respectively; *p* = 0.002) (Fig. 2a).

Next, PBMCs (5 × 10^5^ cells) were stimulated with fixed numbers of exosomes (5.2 × 10^9^ exosomes). Higher IFN-α production was induced by the SLE exosomes than the HC exosomes (median (IQR), pg/mL: 26.83 (1.72–63.68) vs. 0.00 (0.00–0.00), respectively; *p* = 0.005). Even when PBMCs (5 × 10^5^ cells) were treated with up to a 10-fold higher amount of exosomes from HCs (5.2 × 10^10^ exosomes), production of IFN-α was not detected. TNF-α induction tended to be higher by the SLE exosomes than the HC exosomes (median (IQR), pg/mL: 26.83 (15.73–76.70) vs. 8.18 (6.53–17.09), respectively; *p* = 0.056), whereas IL-1β and IL-6 production did not differ between them (Additional file 2: Figure S1).

To test whether the structural integrity of exosomes is important for the immune response, the SLE exosomes were physically disrupted by ultra-sonication. The disrupted SLE exosomes significantly lost the ability to induce cytokine production. Similarly, SLE serum that was depleted of exosomes failed to induce a significant cytokine production either (Fig. 2b).

**Fig. 3:**
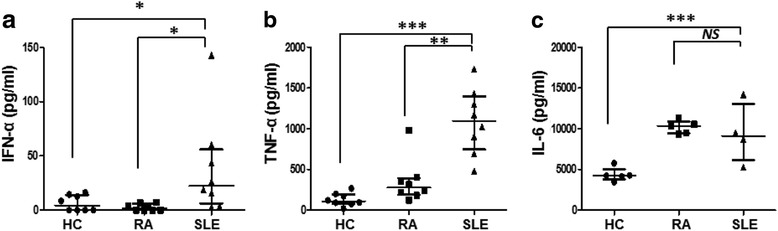
Comparison between cytokine production by rheumatoid arthritis and systemic lupus erythematosus. Production of IFN-α (**a**), TNF-α (**b**), and IL-6 (**c**) by the healthy PBMCs after stimulation with exosomes from healthy controls (*HC*; *n* = 8), rheumatoid arthritis (*RA*; *n* = 8) or systemic lupus erythematosus (*SLE*; *n* = 8) patients were measured. Data are presented as the median and interquartile range. *p < 0.05, **p < 0.01, ***p < 0.0001. *IFN* interferon, *IL* interleukin, *NS* not significant, *TNF* tumor necrosis factor

To assess the possibility of disease-specific effects of SLE exosomes, healthy PBMCs (5 × 10^5^ cells) were treated with 10 μL of exosomes purified from HCs (*n* = 8), rheumatoid arthritis (RA; *n* = 8) or SLE patients (*n* = 8). As compared to the RA exosomes, the SLE exosomes induced PBMCs to produce significantly more IFN-α (median (IQR), pg/mL: 1.84 (0.00–6.07) vs. 22.39 (6.32–55.89), respectively; *p* = 0.013) and TNF-α (median (IQR), pg/mL: 276.2 (188.4–393.0) vs. 1096 (745.3–1396), respectively; *p* = 0.001). IL-6 production did not differ between RA and SLE exosomes (median (IQR), pg/mL: 10331 (9421–10,906) vs. 9097 (6110–13,024), respectively; *p* = 0.556) (Fig. 3).

### SLE exosomes induce cytokine production in a TLR-dependent manner

**Fig. 4:**
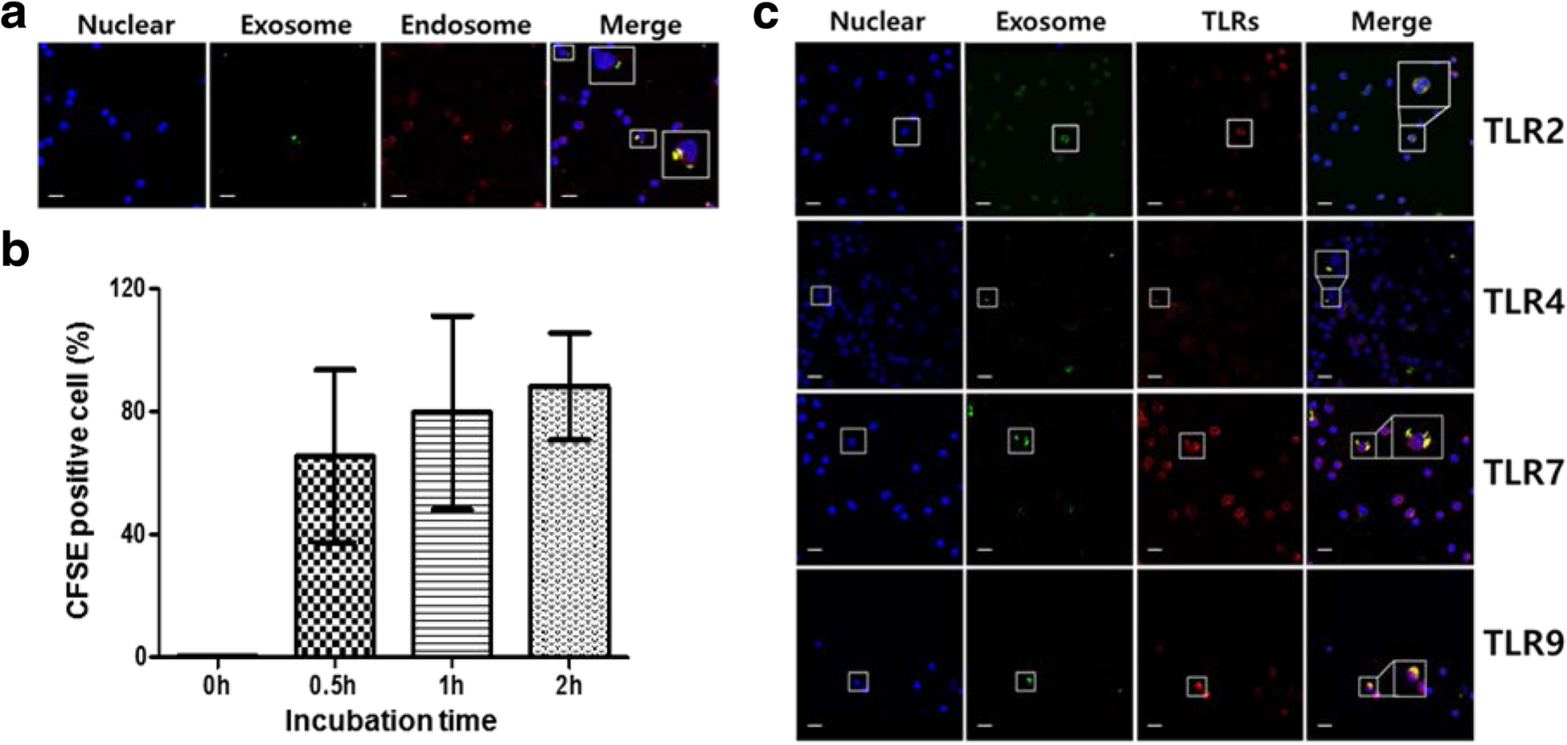
Exosomes from SLE induce secretion of inflammatory cytokines via Toll-like receptors (*TLRs*). **a** Human PBMCs were incubated with carboxyfluorescein succinimidyl diacetate ester (*CFSE*)-labeled SLE exosomes. Confocal images showed that exosomes (*green*) co-localized with endosomes (*yellow* in the merged images). Nucleus (Hoechst blue), endosome (*red*). **b** PBMCs (*n* = 3) were treated with CFSE-labeled SLE exosomes. The percentage of exosome (+) PBMCs increased with time. **c** PBMCs were stained with Hoechst for nucleus (*blue*) and anti-TLR antibodies (*red*). The exosomes (*green*) co-localized with TLRs (*yellow* in the merged images). *Scale bars* = 10 μm

We investigated whether circulating SLE exosomes are engulfed by immune cells. PBMCs were incubated with CFSE-labeled SLE exosomes. After 2 h, the exosomes were observed on the surface and in endosomes of PBMCs (Fig. 4a). The labeled exosomes were visible as early as 30 min after incubation and the exosome uptake increased with time; after 2 h, 88.2 ± 10.0 % cells engulfed the exosomes (Fig. 4b).

**Fig. 5:**
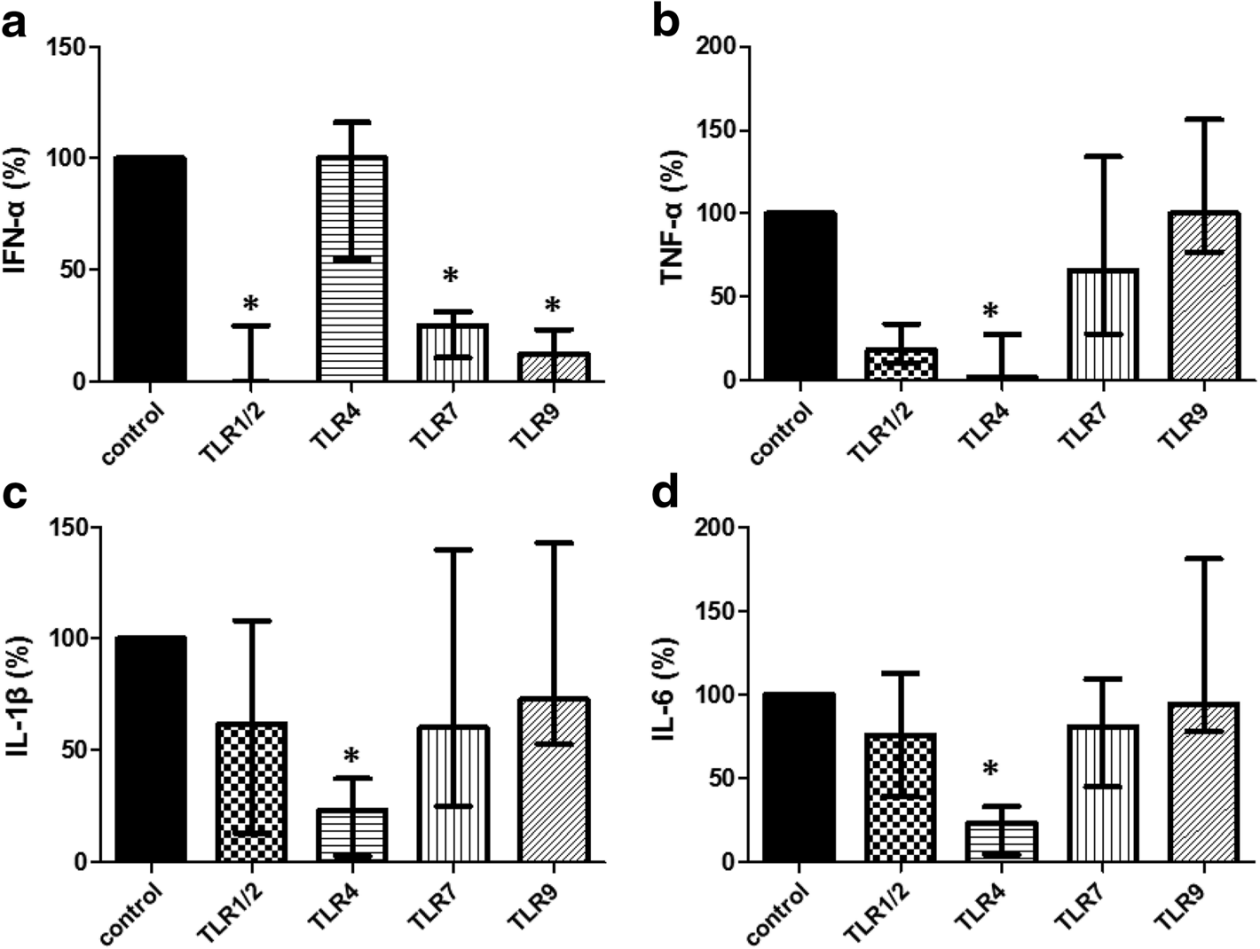
Exosomes from SLE induce secretion of inflammatory cytokines via Toll-like receptors (*TLRs*). Human PBMCs were incubated with SLE exosomes in the presence of specific antagonists to TLR1/2, TLR4, TLR7, and TLR9. After 24 h, levels of IFN-α (**a**), TNF-α (**b**), IL-1β (**c**), and IL-6 (**d**) were determined. Data are presented as the median and interquartile range.*p < 0.05. *IFN* interferon, *IL* interleukin, *TNF* tumor necrosis factor

Next, we tested whether exosomes bind specifically to TLRs on the cell surface and/or reach intercellular TLRs. The exosomes co-localized with TLR2, TLR4, TLR7, and TLR9 (Fig. 4c), suggesting that cytokine production might be mediated in a TLR-dependent manner. Indeed, production of TNF-α, IL-1β, and IL-6 was profoundly decreased in the presence of the TLR4 antagonist, whereas the production of IFN-α was blocked by the TLR1/2, TLR7, and TLR9 antagonists (Fig. 5).

### Serum exosome levels correlate with SLE disease activity

**Fig. 6:**
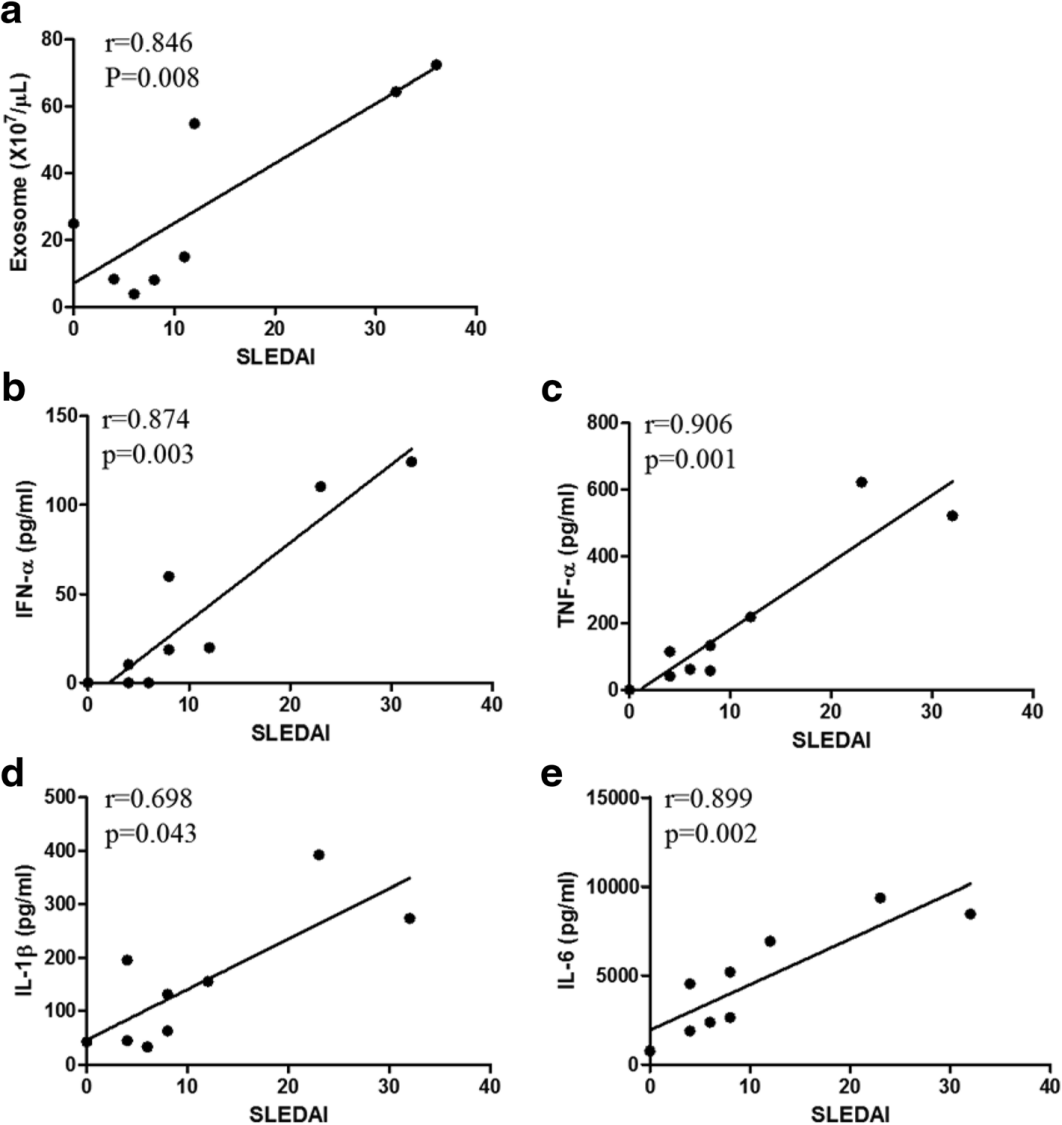
Correlation between SLE disease activity and cytokine production by exosomes. **a** Serum exosome levels correlated significantly with SLE disease activity index 2K (*SLEDAI*) in nine patients with SLE. Production of IFN-α (**b**), TNF-α (**c**), IL-1β (**d**), and IL-6 (**e**) by the healthy PBMCs after stimulation with SLE exosomes (*n* = 8) correlated with SLEDAI. *P* values were generated by using Spearman correlation. *IFN* interferon, *IL* interleukin, *TNF* tumor necrosis factor

We investigated whether higher SLE disease activity was associated with increased exosome levels and proinflammatory cytokine production. The serum exosome levels based on AChE assay significantly correlated with SLE disease activity (Spearman rho = 0.846, *p* = 0.008). In addition, production of IFN-α, TNF-α, IL-1β, and IL-6 by SLE exosomes correlated significantly with SLE disease activity (Fig. 6).

## Discussion

SLE is a systemic autoimmune disease that leads to local and systemic inflammation and damage in multiple organs [19]. Intercellular communication is of paramount importance for both the normal and abnormal immune response. In the current study, we identified exosomes as potential intercellular messengers to promote inflammatory response in SLE; SLE exosomes were able to elicit a significant inflammatory response in a TLR-dependent manner, and the levels of circulating exosomes correlated with disease activity of SLE.

Apoptotic cells in inflamed tissues might release more exosomes into the blood since proper clearance of cell debris is compromised in SLE [20, 21]. Those exosomes can reach and activate immune cells at remote sites via blood circulation. Indeed, increased levels of exosomal miRNA in the urine of patients with active lupus nephritis suggest that inflamed organ or tissue can serve as an important source of exosome production [22]. Therefore, the levels and composition of circulating exosomes in SLE patients might be associated with SLE disease activity.

In the current study, the circulating exosomes from SLE patients were proinflammatory; they were able to induce healthy PBMCs to produce inflammatory cytokines (Fig. 2a). In addition, IFN-α and TNF-α production by a fixed number of exosome particles was higher for the SLE exosomes than the HC exosomes, while IL-6 production per exosome particle did not differ between them (Additional file 2: Figure. S1). Strikingly, exosomes from patients with RA were able to induce IL-6 production but not IFN-α production (Fig. 3). The difference between RA and SLE exosomes in regard to IFN-α production is of particular interest, since type 1 interferon has been postulated as a key cytokine in SLE but not in RA [23–25]. One might speculate that the composition and biologic effects of exosomes are disease-specific. This is supported by the finding that microparticles from patients with active SLE have higher levels of immunoglobulins and complement factors at the expense of the structural proteins [11]. It remains to be defined whether the exosomes carry the disease-specific “molecular signature”, such as the “synovial signature” in RA or “kidney signature” in SLE.

It is striking that the exosomes lost their biological effect when their microvesicular structure was physically disrupted (Fig. 2b). This suggests that not only the amount and composition but also the structural integrity are crucial for their biologic function. Since the cross-linking of receptors on target cells is a common initial step in cell activation, the activating molecules on the exosomes, wrapped spatially tightly together, might better cross-link the receptors on the target cells than in their free form. The membrane structure might allow exosomes to be better engulfed by target cells and so reach intracellular receptors. Indeed, the exosomes from SLE patients were able to induce IFN-α that is mainly triggered by activation of the intracellular endosomal TLR7 and TLR9 [26]. Our findings show that the circulating SLE exosomes can bind to and activate both surface and endosomal TLRs, leading to TLR-mediated proinflammatory cytokine secretion (Fig. 4). Since the blockade of TLR with specific antagonists inhibited the cytokine production, the SLE exosomes exert their biologic function, at least in part, in a TLR-dependent fashion.

The disease activity correlated significantly with the levels of circulating exosomes and their proinflammatory potential, implying that a higher disease activity is associated with both quantitative and qualitative changes in produced SLE exosomes (Fig. 6). Whether the increased exosome production is a cause or result of the increased SLE activity, or both, needs further investigation. Taken together, exosomes in SLE are effective intercellular messengers and possibly contribute to the pathologic immune response [22]. Given the strong immune stimulatory effect of exosomes, removal of the circulating exosomes might offer a novel therapeutic approach in SLE.

The current report has several limitations that might ignite interest for further investigations. First, the cellular source of the circulating exosomes and mechanisms by which exosomes induce inflammatory cytokine production in patients with active SLE need to be defined. Second, the molecules in exosomes that activate the TLRs need to be identified. By characterizing the proteins and DNA/RNA in the SLE exosomes using mass spectrometry and miRNA microarray, the cellular origin of the exosomes could be indirectly identified. Third, since SLE patients with higher SLEDAI received more intense medical treatment, the effects of the concomitant medications on the exosome production and function need to be further examined. Last but not the least, a prospective study with a large sample size is needed to address whether or not the amount and function of exosomes change over time with SLE treatment.

## Conclusions

The circulating exosomes are immunologically active and their levels correlate with disease activity in SLE patients. The circulating exosomes might serve as novel biomarkers of SLE disease activity.

## Additional files

Additional file 1: Table S1. Demographic and clinical characteristics of the SLE patients. (DOCX 16 kb)
Additional file 2: Figure S1. Cytokine production by fixed number of exosomes. IFN-α (A), TNF-α (B), IL-1β (C), and IL-6 (D) production by the healthy PBMCs after stimulation with a fixed number of exosomes from healthy controls (HC) or systemic lupus erythematosus (SLE) patients were measured. Data are presented as the median and interquartile range. (PDF 87 kb)

## Abbreviations

AChE: Acetylcholinesterase
CFSE: Carboxyfluorescein succinimidyl diacetate ester
ELISA: Enzyme-linked immunosorbent assay
HC: Healthy control
IFN: Interferon
IL: Interleukin
IQR: Interquartile range
miRNA: MicroRNA
PAMP: Pattern-associated molecular patterns
PBMC: Peripheral blood mononuclear cell
PBS: Phosphate-buffered saline
RA: Rheumatoid arthritis
SLE: Systemic lupus erythematosus
SLEDAI-2K: SLE disease activity index 2000
TEM: Transmission electron microscopy
TLR: Toll-like receptor
TNF: Tumor necrosis factor
