# Correction to: Circulating exosomes from patients with systemic lupus erythematosus induce an proinflammatory immune response

**DOI:** 10.1186/s13075-020-02206-y

**Published:** 2020-05-08

**Authors:** Joo Youn Lee, Jin Kyun Park, Eun Young Lee, Eun Bong Lee, Yeong Wook Song

**Affiliations:** 1 Department of Molecular Medicine and Biopharmaceutical Sciences, BK 21 plus Graduate School of Convergence Science and Technology, and College of Medicine, Medical Research Institute, Seoul National University, Seoul, Korea; 2 Division of Rheumatology, Department of Internal Medicine, Seoul National University College of Medicine, Daehak-ro, Jongno-gu, Seoul, 03082 Korea

**Correction to: Arthritis Res Ther (2016):18, 264**

**https://doi.org/10.1186/s13075-016-1159-y**

Following publication of the original article [1], the authors would like to update the funding acknowledgement to the following:

**Acknowledgements**

This research was supported by a grant of the Korea Health Technology R&D Project through the Korea Health Industry Development Institute (KHIDI), funded by the Ministry of Health & Welfare, Republic of Korea (grant number: HI14C1277 and HI13C1754), SNUH research grant (number: 0420160770) and Rheumatology Research Foundation (RRF-2016-01).
