# Supplementary material for: Circulating exosomes from patients with systemic lupus erythematosus induce an proinflammatory immune response
Source: Arthritis Res Ther. 2016 Nov 16;18:264. doi: 10.1186/s13075-016-1159-y (PMC5112700; doi:10.1186/s13075-016-1159-y)
Supplement: Additional file 1: Table S1. — Demographic and clinical characteristics of the SLE patients. (DOCX 16 kb) [file 13075_2016_1159_MOESM1_ESM.docx]

**Table S1. Demographic and clinical characteristics of the SLE patients**

|  | SLE patients  (n = 19) |
| --- | --- |
| Age, years, median [IQR] | 39.8 [24.9-52.5] |
| Female, n (%) | 19 (100) |
| SLE duration, years, mean ± SD | 7.3 ± 7.8 |
| ANA, n (%) | 19 (100) |
| ESR, mm/hour, mean ± SD | 35.5 ± 22.9 |
| Anti-dsDNA (mg/dL), median [IQR] | 89.7 [34.8 – 235] |
| C3 (mg/dL), median [IQR] | 53 [32 – 64] |
| C4 (mg/dL), median [IQR] | 6 [6-15] |
| SLEDAI-2K, mean ± SD | 14.3 ± 11.1 |
| Increased DNA, n (%) | 17 (89.5) |
| Low complement, n (%) | 15 (78.9) |
| Fever, n (%) | 5 (42.1) |
| Alopecia, n (%) | 8 (42.1) |
| Hematuria, n (%) | 8 (42.1) |
| Rash, n (%) | 7 (36.8) |
| Mucosal ulcer, n (%) | 6 (31.6) |
| Proteinuria, n (%) | 6 (31.6) |
| Thrombocytopenia, n (%) | 5 (26.3) |
| Leukopenia, n (%) | 5 (26.3) |
| Lupus headache, n (%) | 4 (21.1) |
| Arthritis, n (%) | 4 (21.4) |
| Pyuria, n (%) | 4 (21.1) |
| Seizure, n (%) | 2 (10.5) |
| Pleurisy, n (%) | 2 (10.5) |
| Myositis, n (%) | 1 (5.3) |
| Urinary cast, n (%) | 1 (5.3) |
| Treatment |  |
| CS, n (%) | 19 (100) |
| Prednisolone equivalent, mg/day, median [IQR] | 10 [5-12.5] |
| Hydroxychloroquine, n (%) | 12 (63.2) |
| NSAID, n (%) | 2 (10.5) |
| Cyclophosphamide, n (%) | 2 (10.5) |
| Methotrexate, n (%) | 1 (5.3) |
| Sulfasalazine, n (%) | 1 (5.3) |
| Azathioprine, n (%) | 1 (5.3) |

ANA, anti-nuclear antibody; CS, corticosteroid; ESR, erythrocyte sedimentation rate; IQR, interquartile range; NSAID, nonsteroidal anti-inflammatory drug; SLE, systemic lupus erythematosus; SLEDAI-2K, SLE disease activity index 2000.
