# Supplementary figures and images for: Circulating exosomes from patients with systemic lupus erythematosus induce an proinflammatory immune response
Source: Arthritis Res Ther. 2016 Nov 16;18:264. doi: 10.1186/s13075-016-1159-y (PMC5112700; doi:10.1186/s13075-016-1159-y)

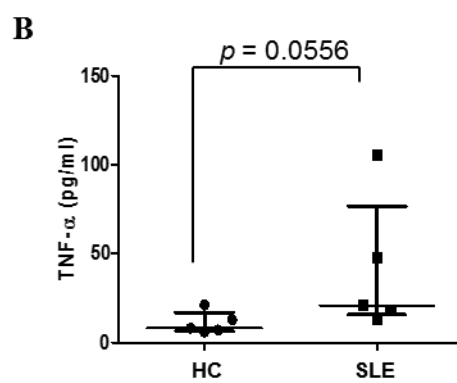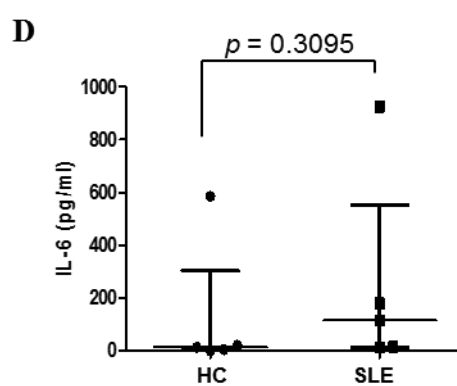

Supplement: Additional file 2: Figure S1. — Cytokine production by fixed number of exosomes. IFN-α (A), TNF-α (B), IL-1β (C), and IL-6 (D) production by the healthy PBMCs after stimulation with a fixed number of exosomes from healthy controls (HC) or systemic lupus erythematosus (SLE) patients were measured. Data are presented as the median and interquartile range. (PDF 87 kb) [file 13075_2016_1159_MOESM2_ESM.pdf]
